# Microplastic hotspots mapped across the Southern Ocean reveal areas of potential ecological impact

**DOI:** 10.1038/s41598-024-79816-y

**Published:** 2024-12-30

**Authors:** Aidan Hunter, Sally E. Thorpe, Arlie H. McCarthy, Clara Manno

**Affiliations:** 1 British Antarctic Survey, High Cross, Madingley Road, Cambridge, UK; 2 Helmholtz Institute for Functional Marine Biodiversity (HIFMB) at the University of Oldenburg, Ammerländer Heerstraße 213, 26129 Oldenburg, Germany; 3 Alfred-Wegener-Institut Helmholtz-Zentrum für Polar- und Meeresforschung, Am Handelshafen 12, 27570 Bremerhaven, Germany; 4 Department of Zoology, University of Cambridge, Downing Street, Cambridge, UK

**Keywords:** Ecology, Ecology, Environmental sciences, Ocean sciences

## Abstract

Marine microplastic is pervasive, polluting the remotest ecosystems including the Southern Ocean. Since this region is already undergoing climatic changes, the additional stress of microplastic pollution on the ecosystem should not be considered in isolation. We identify potential hotspot areas of ecological impact from a spatial overlap analysis of multiple data sets to understand where marine biota are likely to interact with local microplastic emissions (from ship traffic and human populations associated with scientific research and tourism). Then we account for cumulative effects by identifying which areas with potential elevated microplastic-biota interaction are already subject to climate change related stresses (ocean warming and acidification). Our analysis indicates that biologically productive coastal areas in proximity to populated facilities are where microplastics pose most risk to the ecosystem, and that the northern Antarctic Peninsula is likely to be the main risk hotspot. This study is the first to map the threat of microplastics to the Southern Ocean ecosystem in a multi-stressor context, locating where microplastic monitoring programmes and mitigation measures may be considered a matter of urgency.

## Introduction

Marine microplastics pose risk to organisms and may perturb ecological processes^1^ throughout the world’s oceans^2^, including remote and isolated regions such as the Southern Ocean (SO)^3^. Due to remoteness and difficulties conducting research in extremely seasonal environments, there have been relatively few microplastic sampling campaigns in the polar oceans, and the SO is the least studied region. Measurements from most SO studies confirm the presence of microplastic, though at low concentrations compared with other seas^4^. Nonetheless, if the global trend of rising plastic manufacture and disposal continues as expected^5^ then SO microplastic will likely become increasingly prevalent. Generating baseline information on the prevalence and sources of SO microplastic, its interactions with the ecosystem, and whether it exacerbates other environmental stresses therefore requires prompt investigation before pollution levels increase further.

Microplastic in the SO may originate from local sources^6,7^ or from distant lower latitude seas^8^, with meridional transport across the Antarctic Circumpolar Current potentially facilitated in part by mesoscale eddies^9,10^. Local emission sources of microplastic include ship traffic and Antarctic research facilities. Measured as total ship-days at sea, SO shipping activity consists mostly of fishing and tourist vessels, followed by research and resupply ships^11^. Lost or discarded fishing gear, a significant proportion of marine plastic worldwide^12^ and within the SO^13^, results in animal entanglement and “ghost fishing”, and gradually degrades to produce microplastics^14^. Shipping also produces microplastics and synthetic particles directly via hull degradation and wastewater^15,16^. As the quantity of expelled wastewater and microplastic is proportional to ship population size, it is likely that highly populated tourist vessels produce most of this pollution^17^. The Antarctic is very sparsely populated: numerous research facilities distributed around the continent house approximately 5000 people throughout austral summer^18^. Wastewater from these facilities can contain microplastic^19^, so each one is a potential emission source. Emission rates partly depend on the facility’s population size and wastewater disposal protocols, which vary from comparable to municipal water treatment to considerably less stringent^20,21^. Although microplastic concentrations may be minimal at the scale of the entire SO, they are significant in proximity to emission sources, particularly in coastal areas nearby research facilities, where they may pose ecological risk^22^.

The SO is highly productive during the summer months, with large and intense phytoplankton blooms associated with marginal sea ice zones, shelf seas, and nutrient-rich upwelling areas in the open ocean^23,24^. This supports massive biomass of Antarctic krill (*Euphausia superba*, hereafter krill), a key species that supports myriad predators including fish, birds, and migrating cetaceans, and a krill fishery^25^, and also plays a fundamental role in the biogeochemical cycle^26,27^. Krill have been observed ingesting microplastic both in laboratory settings^28^, with impacts on their physiology and behaviour^29,30^, and in their natural environment^31^ where the impacts are understudied.

Microplastic pollution in the SO occurs concomitantly with other environmental changes^32,33^. Considered a “bellwether” for ocean acidification^34^, the SO is subject to some of the most rapid declines in surface ocean pH and shoaling of the carbonate saturation horizon. Model studies and experiments on the impact of elevated CO_2_ concentrations ($\hbox {pCO}_2>1000$ $\mu \text {atm}$, corresponding to the 100–300 year forecast of the RCP 8.5 scenario) on krill indicated negative impacts on early life stages^35^. Significant trends in sea surface temperature have also been observed in recent decades: much of the Indo-Pacific sector, the western Antarctic Peninsula, and South Georgian waters have warmed^36,37^, while the eastern Pacific sector, western Indian Ocean sector, and east Antarctic coastal waters have cooled^38,39^. Analyses of krill distribution data indicate that ocean warming, together with reductions in sea ice, have likely caused a southward contraction of the krill population distribution in the southwest Atlantic sector of the SO^40^.

Krill potentially have heightened vulnerabilities to multi-stressor environments due to their physiology, behavioural traits, and the rapid environmental changes of their habitats^41^. Studies on how krill respond to the combination of rising temperature and declining pH revealed that krill compensated for lowered pH within hours to days, and that their longer-term growth and survival were diminished mostly by rising temperature^42,43^. Microplastic exposure may adversely impact the robustness of Antarctic species to environmental change, exacerbating existing stresses^41^. A study of the combined impacts of low pH conditions and nanoplastic exposure showed more suppressed development of krill embryos under the combined stresses than when considered separately^44^. Thus, to understand realistic microplastic toxicity thresholds and potential trophic cascade impacts through the pelagic food web, this pollutant should be considered within the context of other local environmental changes, not in isolation.

In this study, we perform a spatial overlap analysis to identify and map multi-stressed regions in the SO where lower trophic level biota, and in turn, the wider ecosystem is at greatest potential risk from microplastics. We focus on krill as a threatened organism, known to consume microplastic in its natural environment with potential deleterious impacts, because of its fundamental role in the SO food web. We locate local potential sources of microplastic emissions from populated Antarctic facilities and commercial shipping and research vessels. By comparing distributions of potential emissions and biota, we determine where risk of direct interaction with microplastic is greatest. Then, to complete the microplastic risk map, we account for cumulative impacts by identifying which areas with elevated microplastic-biota interaction are already subject to climate change related stresses (i.e., ocean warming and acidification). To easily visualise these potential “microplastic hotspots”, we have produced an online interactive map as a tool to support future targeted microplastic research, monitoring programmes, and actions to mitigate the risks associated with this pollutant.

## Data

### Biota prevalence

#### Antarctic krill

**Fig. 1:**
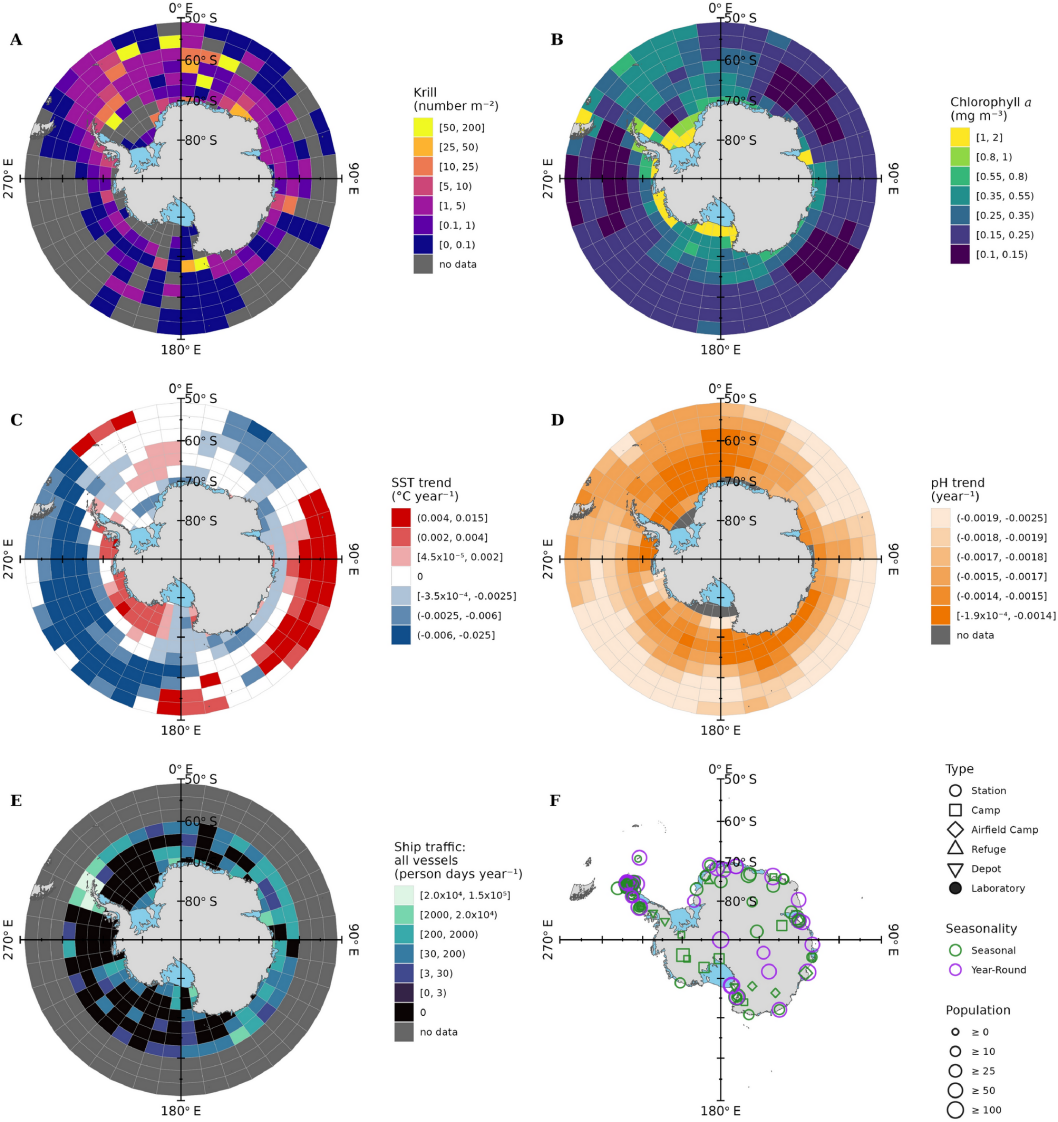
Data used to create risk maps. (**A** and **B**) are biota metrics: the interannual mean density of krill compiled from historic net samples (1926–2016), and the interannual mean chlorophyll *a* concentration in surface waters from satellite observations (1997–2007), both representing only the months January–March. (**C** and **D**) are abiotic stress metrics: the linear rates of change of sea surface temperature (1982–2021) and pH (1985–2021). (**E** and **F**) are human activities: interannual mean ship traffic intensity from Automatic Identification System records (2014–2018), and locations and properties of terrestrial facilities. Note that panels (**A**, **B**, and **E) **display data on a log scale. Maps made with R^48^ packages ‘sf’^49^ and ‘ggplot2’^50^, using Natural Earth free vector and raster map data.

Scientific net samples spanning 1926–2016 informed the spatial distribution of krill^45^. These data included the original measurements of krill density $\bigl ($individuals $\hbox {m}^{-2}\bigr )$ and refined estimates that standardised the measurements by depth, time of year, and type of net. Using the standardised estimates, we calculated krill densities following the method of Atkinson *et al.*^46^, modified only to account for skewed measurement distributions by using the geometric — rather than arithmetic — mean of krill catches from multiple net samples within $9^\circ$ longitude $\times$ $3^\circ$ latitude grid cells (Fig. 1A). Measurement zeros were assimilated into the geometric mean using the method of Habib^47^. The grid cell size used for krill determined the spatial resolution of our maps, as all other data were identically gridded.

**Fig. 2:**
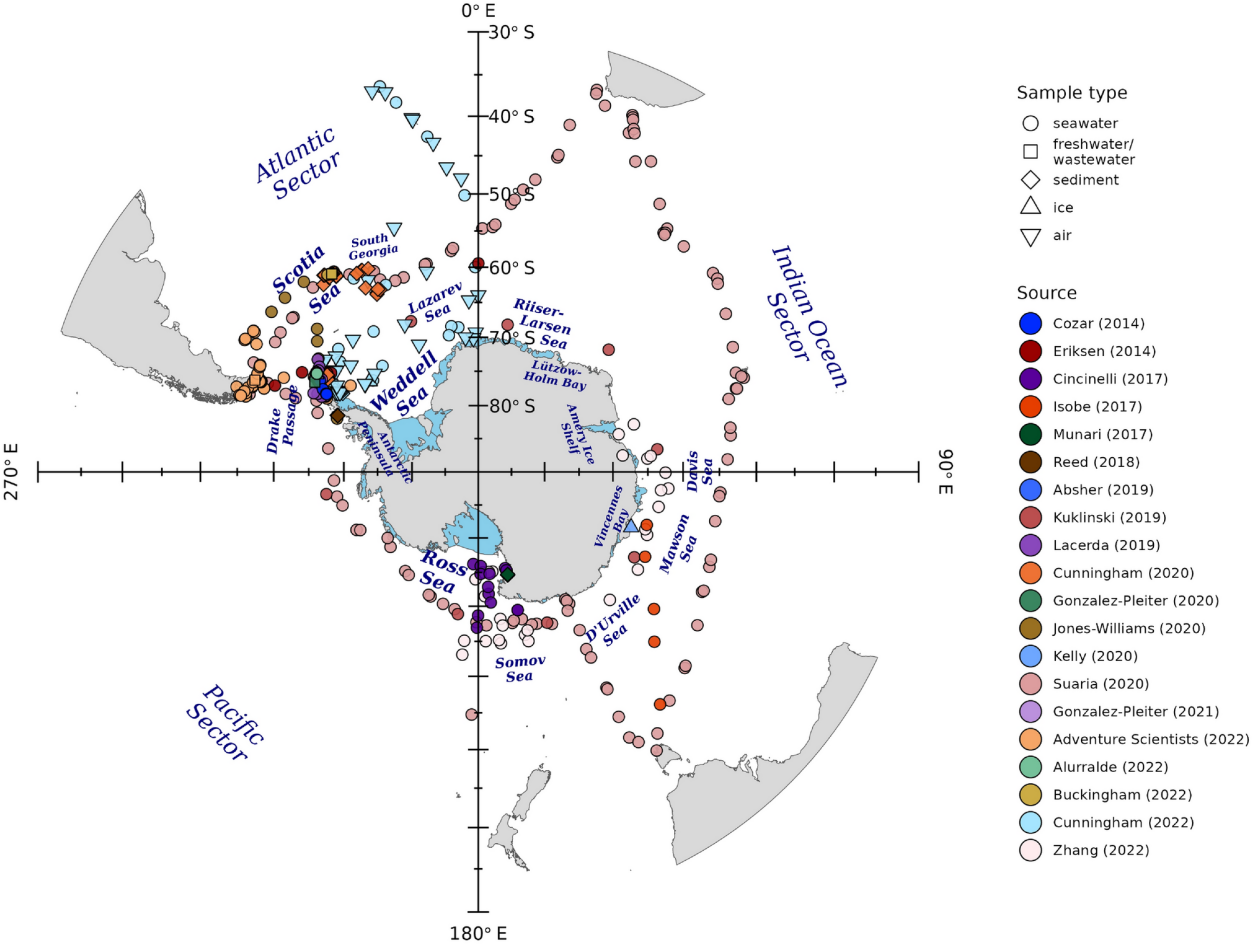
Southern Ocean plastic samples collected between 2010 and 2022. More detail and references are provided in Table S1. See the interactive web app^69^ for measurement values and metadata associated to each sample. Map made with R^48^ packages ‘sf’^49 ^and ‘ggplot2’^50^, using Natural Earth free vector and raster map data.

The resulting krill distribution map was patchy where samples were lacking, particularly in the offshore regions $80^\circ$–$140^\circ \,$W (north of $65^\circ \,$S) and $80^\circ$–$150^\circ \,$E (north of $60^\circ \,$S), as well as relatively small areas within the Weddell Sea and eastern Ross Sea (Fig. 1 A; refer to Fig. 2 for place names). Krill were observed in greatest abundance $\bigl (>10$ individuals $\hbox {m}^{-2}\bigr )$ most often in waters within $50^\circ \,$W–$50^\circ \,$E, both in southern waters close to the Antarctic coast and as far north as $55^\circ \,$S. Moderate abundances $\bigl ($from 1 to 10 individuals $\hbox {m}^{-2}\bigr )$ were evident all around the Antarctic continent within $6^\circ$–$9^\circ$ latitude from the coast. Further offshore, abundances were lower, except for within the Scotia Sea and eastwards to the Riiser-Larsen Sea where moderate–high abundance was observed as far north as $50^\circ \,$S.

#### Chlorophyll *a* concentration

Phytoplankton were visualised using chlorophyll *a *concentration data derived from ocean colour satellite observations^51^. This data product provided chlorophyll *a* concentration estimates for the surface 5 m at high spatial resolution ($1/12^\circ$ longitude $\times$ $1/12^\circ$ latitude), and employed modelling methods^52,53^ to group total chlorophyll *a* into three phytoplankton size classes (pico, nano, and micro scales). The data were available as monthly means over a ten year period (1997–2007), representing typical phytoplankton distributions throughout the year. We mapped these high resolution data onto a $9^\circ$ longitude $\times$ $3^\circ$ latitude grid by calculating geometric mean values within grid cells (Fig. 1 B). We used the mean chlorophyll *a* concentration measurements from January–March to align with the in situ krill data, of which the majority were collected during these months.

Surface chlorophyll *a* was most concentrated $\bigl (\ge 0.8$ mg $\hbox {m}^{-3}\bigr )$ around the western coast of the Antarctic continent, at the ice sheet edges in the Ross Sea, Weddell Sea, and along the Antarctic Peninsula (Fig. 1 B). Along the eastern Antarctic coast these high concentrations were confined to the edge of Amery Ice Shelf edge in Prydz Bay. Moderate concentrations $\bigl ($from 0.35 to 0.8 mg $\hbox {m}^{-3}\bigr )$ were observed between the tip of the Antarctic Peninsula and the eastern Scotia Sea, as far north as South Georgia; in the Ross Sea, offshore to around $65^\circ \,$S; and in coastal waters east of Amery Ice Shelf. Elsewhere, far offshore from the western Antarctic Peninsula and around most of the eastern side of the continent, observed chlorophyll *a* concentrations were relatively low $\bigl (< 0.25$ mg $\hbox {m}^{-3}\bigr )$.

### Abiotic trends

#### Sea surface temperature

A global sea surface temperature data product^54^, generated from analysis of satellite observations^55,56^, has been used by Good *et al.*^57^ to map temperature trends during 1993–2021. The satellite data product provided daily measurements of sea water temperature at 20 cm depth, spanning 1981–2022, and spatially resolved to $0.05^\circ$ longitude $\times$ $0.05^\circ$ latitude. For our analysis, we recreated the map of Good *et al.*^57^ to include the full time period of the available observations. We extracted the satellite data^54^ for areas south of $45^\circ \,$S then generated a map of sea surface temperature trends during 1982–2021 (years with complete records) following the five-step method used by Good *et al.*^57^. (1) Temporal resolution was reduced by calculating monthly means within each $0.05^\circ$ $\times$ $0.05^\circ$ grid cell, then spatial resolution was reduced by calculating mean values within each of our ($9^\circ$ longitude $\times$ $3^\circ$latitude) map grid cells. (2) Monthly means were averaged over the period 1993–2014 to generate a climatology. (3) Differencing the climatology from the monthly means produced temperature anomalies. (4) The temperature anomaly time series were passed through the X11 seasonal adjustment process^58,59^ to decompose the data into seasonal and trend components. (5) Gradients $\bigl ({^\circ \hbox {C}} \;\hbox {year}^{-1}\bigr )$ of trends were calculated from linear models, that also provided confidence intervals around gradients. The resulting map of sea surface temperature trends is displayed in Fig. 1 C, where regions with statistically insignificant $(p>0.05)$ trends are shown as white.

Sea surface temperature trends had uneven spatial distribution; both the magnitude and direction of trends varied with location (Fig. 1 C). The greatest long term rises in temperature $\bigl ($between 0.004 and 0.015 ${^\circ \hbox{C}}$ $\hbox{year}^{-1}\bigr )$ were observed far offshore ($50^\circ$–$60^\circ \,$S) from the Antarctic continent in the Indian Ocean ($65^\circ$–$140^\circ \,$E) and western Pacific ($160^\circ \,$E–$170^\circ \,$W) sectors of the SO, and north of South Georgia. More moderate warming trends $\bigl (<0.004$ ${^\circ \hbox{C}}\; \hbox {year}^{-1}\bigr )$ were observed along the western side of the Antarctic Peninsula and in the northeast Weddell Sea. Elsewhere, in 73% of the ocean area south of $50^\circ \,$S, the observed long term trend in sea surface temperature was either negative or non-significant.

#### Sea surface pH

Time series of global sea surface pH estimates were sourced from a data product^60 ^produced using a feed-forward neural network to assimilate data ensembles^61^. These estimates of monthly mean pH spanned 1985–2021 and were spatially resolved to $1^\circ$ longitude $\times$ $1^\circ$ latitude. We created a map of linear trends in pH (Fig. 1 D) following the same five-step method used to map sea surface temperature trends. The only differences were that the pH data had lower spatial resolution and were already provided as monthly means.

Long term trends in surface water pH were significantly negative throughout the entire SO (Fig. 1 D). The greatest negative trends in pH $\bigl ($between $-0.0019$ and $-0.0025$ $\hbox {year}^{-1}\bigr )$ were far from the Antarctic coast, north of $60^\circ \,$S in the Indian Ocean and Pacific sectors of the SO.

### Local human activity

#### Ship traffic

Automatic Identification System (AIS) ship traffic data spanning 2014–2018 were used to estimate ship time in the SO. These data, provided by Lloyd’s List Intelligence (LLI), were the same used by M^c^Carthy *et al.*^62^ in their study of invasive species risks posed by SO shipping, which provides a full description of the AIS data processing. Instead of grouping ship traffic data into the ecoregions defined by Spalding *et al.*^63^, as M^c^Carthy *et al.*^62^ did, we calculated the time each vessel spent within each of our map grid cells. For comparability with data on facility population size, we converted the unit of days $\hbox{ship}^{-1}$ $\hbox {year}^{-1}$ into person days $\hbox {ship}^{-1}$ $\hbox {year}^{-1}$ by multiplying the time each vessel spent in a grid cell by the typical number of people on board that vessel^64–66^. The AIS data were grouped by vessel type (fishing, tourist, cargo/supply, research, other), so summing ship time within these groups then taking the mean across years produced five maps of mean ship time, one for each vessel type (Supplementary Fig. S1). Summing over vessel types produced a map of mean total annual ship time (Fig. 1 E).

Ship traffic in grid cells around the tip of the Antarctic Peninsula varied between $2\times 10^4$ and $1.5\times 10^5$ person days $\hbox {year}^{-1}$, approaching a maximum ship traffic density of 2 person days $\hbox {year}^{-1}$ $\hbox {km}^{-2}$, greatly exceeding all other regions. The northern Antarctic Peninsula is a hotspot for fishing and tourism, and also hosts more research vessels than elsewhere in the SO due to the high density of facilities (Supplementary Fig. S1).

#### Antarctic facilities

Data on Antarctic research stations and facilities were sourced from The Council of Managers of National Antarctic Programs^67^. This included location, facility type (research station, camp, airfield camp, refuge, depot, or laboratory), seasonality of operation (year-round or seasonal), and peak population size (Fig. 1 F). Peak population size of facilities varied between 0 and 1200, approximately following a log-normal distribution. The most populous facility, M^c^Murdo research station, was an outlier; the next largest facility, Syowa research station, had peak population of 170. We assumed that year-round facilities operate at peak population, *p*, all year so that person days $\hbox {year}^{-1}=365\,p$, whereas seasonal facilities were assumed to operate for six months per year so that person days $\hbox {year}^{-1}=182.5\,p$. We grouped facility population sizes into five categories: 0–9, 10–24, 25–49, 50–99, and $\ge 100$.

Potential marine emissions from facilities depends on their population size, distance from the coast, and density. There were six facilities with $p>100$ people and situated within 10 km of the coast: on the Antarctic Peninsula there is Rothera Research Station, Base Marambio, and Base Presidente Eduardo Frei Montalva; at $40^\circ \,$E on the eastern side of Lützow-Holm Bay there is Syowa Station; at $110^\circ \,$E on the eastern side of Vincennes Bay there is Casey Station; and on Ross Island in the western Ross Sea there is M^c^Murdo Station. Each of these large stations has one or more smaller facilities in close proximity, and there is another large cluster of facilities (comprising two small stations and four of population size ${\ge 50}$) at $10^\circ \,$W–$15^\circ \,$E on the Fimbul Ice Shelf.

### Microplastic samples

Microplastic measurements from the SO (Table S1) are spatially heterogeneous, with large areas devoid of samples (Fig. 2). Most sampling has targeted the Atlantic sector, usually around the northern Antarctic Peninsula, though also concentrating in South Georgian waters. Spatial coverage appears fairly comprehensive in the western Atlantic sector, from Drake Passage to the Lazarev Sea, but these data span numerous years so intra-annual spatial coverage is minimal and in some areas, notably the Weddell Sea, samples derive from a single sampling campaign. Spatial data gaps are far larger within the Pacific and Indian Ocean sectors, where sampling has focussed on the Ross and Somov seas, and, to a lesser extent, the coastal waters westward from there to the Davis Sea. Due to limited spatio-temporal coverage and inconsistent sampling designs that hinder between-study microplastic abundance comparisons, we did not use these data to derive risk maps. The microplastic data are mapped in Fig. 2 to show where sampling has been conducted and to determine, with reference to our environmental risk maps, how this corresponds to areas of concern.

The data include microplastic concentration and presence/absence measurements from surface seawater, riverine and glacial freshwater, wastewater from facilities, seabed sediments, sea ice, and air (Fig. 2 and Table S1). Most common, by far, were measurements from surface seawater. Known to be confounded by other variables such as weather conditions, sea surface measurements are unreliable indicators of ambient microplastic concentrations and input rates from local or remote sources^8^. More direct information on marine microplastic input rates from some local sources come from measuring concentrations in freshwater, wastewater, and air, but few SO sampling campaigns have collected these data. Equally scarce were samples from ice, a medium that could be important to study as glacial ice is another potential source of marine microplastic and sea ice may act as a microplastic transport mechanism and transient sink/source by trapping sea surface particles during ice formation then releasing them elsewhere while melting^68^. Sediment samples collected near the Antarctic Peninsula, South Georgia, and the Ross Sea, prove the presence of microplastics on the seabed and, though deposition rates are difficult to determine, may provide information on the location of microplastic sinks. The microplastic data may be explored in more detail, compared to the biotic and abiotic data, filtered, and downloaded via our interactive web app^69^. As our interactive map is dedicated to analyses of microplastic and its potential to impact the ecosystem, it extends the functionality of the existing SOOSmap^70^.

## Methods

**Fig. 3:**
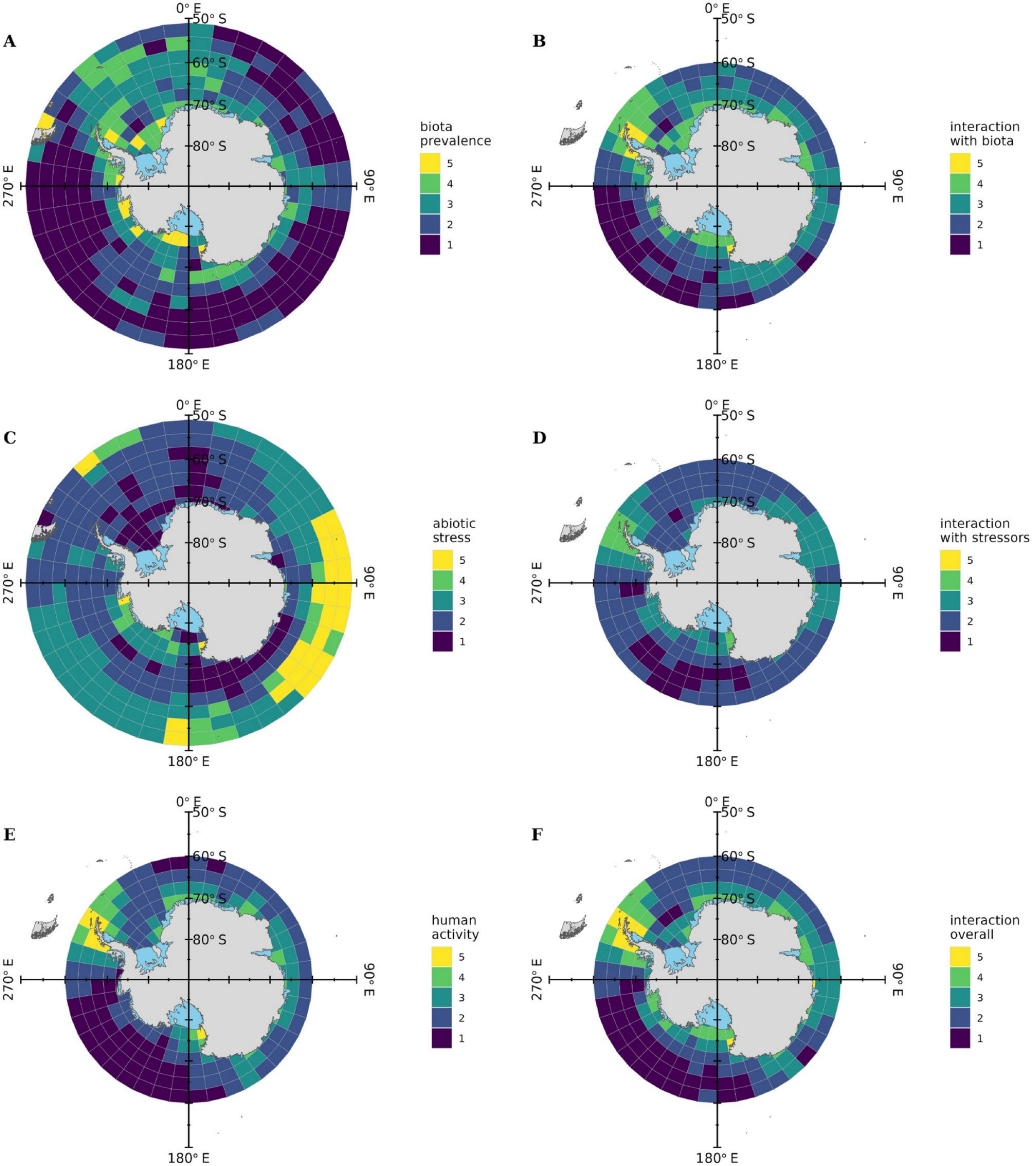
**(A**, **C**, and **E)**: maps of biota (chlorophyll & krill), abiotic stresses (SST & pH trends), and human activity (shipping & facilities) coloured by ranks described in main text. (**B** and **D)**: interaction maps displaying where potential microplastic emission sources overlap with biota and abiotic stresses. (**F)**: combined interaction map displaying where potential microplastic emission sources overlap with biota and abiotic stresses. Maps made with R^48^ packages ‘sf’^49^ and ‘ggplot2’^50^, using Natural Earth free vector and raster map data.

Observed spatial distributions of biota (chlorophyll *a* concentration and krill density), temporal trends in ocean properties (sea surface temperature and pH), and human activity (ship time and terrestrial facilities) were mapped with a resolution of $9^\circ$ longitude $\times$ $3^\circ$ latitude (Fig. 1). These data were used to create risk maps indicating where potential local microplastic emissions are most likely to directly interact with biota, exacerbate existing stresses, and pose greatest environmental risk (Fig. 3). Grid cells of the risk maps were ranked as functions of biota abundance, magnitude of trends in ocean properties, and human presence as a proxy for microplastic emissions. All analyses were performed using R 4.4.1^48 ^and MATLAB 2023b^71^: the names and versions of all packages used are listed in the code repository.

### Discrete ranking scales

The various data were measured with different units so were not directly comparable. To permit comparisons we associated each data set to a discrete ranking scale, from 1 (low) to 5 (high), grading each mapped grid cell from least to greatest concern. Data sets with skewed distributions were transformed to approximate Gaussian distributions, then rankings for each data set were delineated by ascribing four threshold values evenly spaced between the minimum and maximum measurements (Figs. S2 to S4). This simple ranking method made minimal assumptions and was, mostly, consistent between the different data sets.

The distributions of krill density and chlorophyll *a* concentration measurements were right-skewed, and the krill data contained zeros from samples lacking animals as well as numerous blank grid cells where no sampling had taken place. Zeros and outlying values were omitted, then ranking thresholds were set at evenly spaced intervals across the log-transformed measurement range (Supplementary Fig. S2). All grid cells associated to a measurement of zero were assigned a rank of 1 and outlying values were assigned either the minimum or maximum rank.

The discrete ranks ascribed to sea surface temperature and pH measurements represent, respectively, the magnitude of positive and negative linear rates of change. Trends in sea surface temperature were positive or negative depending on region (Fig. 1 C), so the distribution of positive trends was right-skewed. There was no such symmetry in pH trend measurements, which, at this coarse spatial resolution, were all negative (Fig. 1 D). Once again, after omitting outlying measurements, ranking thresholds were set at evenly spaced intervals across the measurement ranges, this time using a square root transform to normalise the distribution of positive sea surface temperature trends (Supplementary Fig. S3).

The influence of terrestrial facilities upon mapped grid cells was ranked by calculating the population density within each cell. For grid cells containing facilities, this was calculated as the total population housed in those facilities divided by the grid cell area. To reduce the impact of arbitrariness in grid cell size and to account for potential dispersion of microplastic, albeit entirely uniform and undirected dispersion, the effective population size in all other grid cells was also calculated. This was done by calculating areas of circles centred on facilities and intersecting grid cell centroids. For each facility-grid cell pair, the distance separating the facility and grid cell defined a radius, then we calculated the circular area and the facility-specific population density $\bigl (\hbox{people}$ 10,000 $\hbox {km}^{-2}\bigr )$ within this area. Multiplying by the ratio of grid cell area to total circular area gives the population density within the grid cell that is attributable to the facility. The effective population density within the grid cell is then found by summing these metrics over all facilities. This method of calculating effective population densities at distance from point-locations where populations are centred is essentially a smoothing operation that models effective population as an inverse square of combined distances from all population point-locations. Rankings for each grid cell were then defined, as above, by specifying evenly spaced intervals between the range of log-transformed population densities (Supplementary Fig. S4). Ship traffic observations were ranked for all grid cells using an identical method (Supplementary Fig. S4).

### Baseline ranks

To identify regions where biota are prevalent and where ocean properties have changed, we calculated and mapped baseline ranks for these two factors (Fig. 3 A, C). These were termed baseline ranks because they represent the spatial distributions of biota and abiotic stresses before considering potential interactions with local microplastic emissions. Baseline ranks were calculated for each mapped grid cell simply as the mean rank across the distinct groups of variables. The baseline map for biota prevalence (Fig. 3 A) was calculated as the mean rank of krill and chlorophyll *a* (Supplementary Fig. S2), while baseline abiotic stress (Fig. 3 C) was the mean rank of sea surface temperature and pH trends (Supplementary Fig. S3). The extent of potential microplastic emissions was similarly represented (Fig. 3 E) as the mean rank of disturbance from ship traffic and facilities (Supplementary Fig. S4), though in this case the latitudinal extent was restricted to $60^\circ \,$S due to the lack ship traffic data further north.

### Potential interactions with microplastic

The potential for local microplastic emissions to interact with biota and abiotic stresses was represented by two maps (Fig. 3 B and D) displaying, respectively, the mean ranks of potential emissions and biota prevalence (Fig. 3 A, E), and the mean ranks of potential emissions and abiotic stresses (Fig. 3 C, E). The main result, a map of combined interactions showing where potential microplastic emissions overlap both with biota and abiotic stresses (Fig. 3 F), was calculated as the mean of the individual interaction maps (Fig. 3 B, D). This combined interaction map of “microplastic hotspots” highlights regions of concern where potential microplastic emissions are most likely to interfere with biota both directly and via exacerbating existing abiotic stresses.

## Results and discussion

The map synthesised from chlorophyll *a* and krill observations displays the joint distribution of phytoplankton and krill (Fig. 3 A). Where krill data were lacking, this planktonic biota prevalence map was defined entirely by the chlorophyll *a*, which was most prevalent in the west Antarctic coastal waters. When compared to the phytoplankton distribution map, it is clear that the high abundance of krill in the Atlantic and Indian Ocean sectors of the SO increased the relative biota prevalence in these areas (Supplementary Fig. S2). More specifically, the biota prevalence ranks indicate greatest planktonic abundance (rank 5) in nearshore waters around western Antarctica, close to the Ross Sea and Weddell Sea ice shelves and along the Antarctic Peninsula (Fig. 3 A). Moderate to high biota prevalence (rank 3–5) was evident in most waters nearshore to the Antarctic continent, up to $6^\circ$ latitude from the coast. Biota prevalence declined further offshore, with the exception of the $50^\circ \,$W–$20^\circ \,$E region spanning the Scotia Sea eastwards to the Lazarev Sea and, to a lesser extent, the ice edge-associated Ross Sea and Davis Sea.

The abiotic multi-stressor map, synthesised from sea surface temperature and pH data, illustrates the joint distribution of long term trends in these two variables (Fig. 3 C). As long term trends in temperature across most of the SO were either insignificant or negative, and assumed low risk, the joint spatial distribution of abiotic stresses most closely followed the pattern of long term pH decline (Supplementary Fig. S3). Due largely to widespread pH declines, almost all waters north of $60^\circ \,$S throughout the Pacific and Indian Ocean sectors of the SO were subject to moderate to high (rank 3–5) abiotic stress, that was maximised where these pH declines coincided with rising temperatures at $65^\circ$–$145^\circ \,$E. Other regions with moderate to high abiotic stress included the southwestern Antarctic Peninsula and the waters surrounding South Georgia. Elsewhere in the Scotia Sea, and in the Weddell, Lazarev, and Somov seas, there was minimal long term abiotic stress (rank 1–2).

Abiotic stress tended to increase with distance from the Antarctic coastline, so the multi-stressor spatial distribution was approximately inverse to the biota prevalence distribution, with the notable exceptions of the southwestern Antarctic Peninsula and waters surrounding South Georgia (Fig. 3 A, C). This suggests that the moderate long term changes to temperature and pH in these two regions may have greater net impact upon SO phytoplankton and krill than the more substantial and widespread changes observed elsewhere.

Ship traffic was concentrated in proximity to terrestrial facilities (Supplementary Fig. S4), so these two data sets reinforced each other when synthesised into the distribution map of human activity, our proxy for local microplastic emissions (Fig. 3 E). The northern Antarctic Peninsula, where human activity concentrates (rank 5) around 48 of the total 112 Antarctic facilities, is the main source of potential microplastic emissions. Due to the relatively large population in this region, there was considerable (rank 4) human activity far offshore in the Scotia Sea and Drake Passage. Elsewhere, this level of human activity was observed only within grid cells bordering the Antarctic coast in proximity to numerous and large facilities including M^c^Murdo (the only other rank 5 grid cell), Syowa, and a cluster of research stations surrounding the prime meridian.

Spatial overlap between human activity and the distributions of biota and abiotic stresses (Fig. 3 B, D) indicate where planktonic biota are most likely to interact with potential local microplastic emissions, and where these emissions have the greatest potential to exacerbate impacts of other environmental changes. The distributions of biota prevalence and human activity are broadly similar (Fig. 3 A, E), with substantial overlap (of areas ranked 3–5) around the Antarctic coastline and Scotia Sea. These areas have most potential for planktonic biota to directly interact with local microplastic emissions. Predictably, due to relatively high human population density and proximity to productive coastline, the main potential hotspots (rank 5) of direct biota-microplastic interaction are the waters surrounding the northern Antarctic Peninsula and near the large M^c^Murdo research station in the Ross Sea. Human activity and biota prevalence diminish north of $65^\circ \,$S so the interaction risk is minimal (rank 1–2) in most of this area, with the exception of the Scotia Sea.

The dissimilarity of the abiotic stress and human activity distributions (Fig. 3 C, E) suggests that, at least in proximity to their sources, local microplastic emissions have limited potential to exacerbate existing stresses upon biota (Fig. 3 D). Given the more substantial overlap between planktonic biota and human activity, it is this direct interaction potential that controls the distribution of overall risk that local microplastic emissions pose to the SO environment (Fig. 3 F).

The derivation of our risk maps did not include the compilation of available microplastic data because it is unsuited to assessing trends or patterns in concentration. This is partly due to the patchy spatio-temporal coverage resulting from minimal, often opportunistic, SO sampling. Furthermore, the data collected by multiple studies suffer from a lack of cohesion and standardisation due to variability in sampling equipment^72^, laboratory identification procedures^73,74^, control data protocols^75^, and even the nomenclature used to report measurements^76^. We can, however, compare the distribution of the available microplastic data to the areas of potential ecological impact identified by our risk maps to assess the overlap between past sampling and areas of concern.

It is encouraging that what we identified as the main hotspot of potential microplastic impacts on the SO ecosystem, the northern Antarctic Peninsula, is where data collection has been most concentrated. We also identified as a hotspot for microplastic impacts, the western Ross Sea, the focus of Pacific sector sampling effort. Spatial correlation between microplastic sampling effort and hotspot areas of concern is unsurprising. Research expeditions often target areas of high biological productivity and/or the waters in proximity to research stations, which are potential microplastic sources and a locus for ship traffic, another microplastic source. We should note that evaluating transport of local microplastic emissions via ocean currents^68 ^and sea ice^77^ was outside the scope of our study, which located potential microplastic sources but not sinks. Sink areas subject to relatively low human activity may, therefore, pose greater risk to biota from microplastic than indicated. Furthermore, we considered only local sources of microplastics in the SO and the distribution of their potential impacts on the ecosystem, but it is also important for monitoring and management that future studies consider input and distribution of microplastics from distant sources^78,79^.

Remoteness of the SO makes monitoring the quantity and impacts of microplastic challenging and costly, so maximising the value of limited samples is important. In this study we created an interactive online web application^69^ that enables further detailed assessment of the available data and identification of areas where microplastic pollution potentially poses ecological risk because the ecosystem is already subject to other environmental stresses and/or there is high risk of biota interacting with microplastic. Microplastic pollution in the SO is expected to intensify, together with other environmental stresses such as ocean acidification and warming^80^. Therefore, focussing future microplastic research effort on hotspot areas of ecological impact together with the adoption of consistent sampling locations is an important step towards creating standardised and statistically rigorous time series survey data that may be used for long-term monitoring and target mitigation measures.

## Supplementary Information

Supplementary Information.

## Data Availability

The ship traffic dataset analysed during the current study are not publicly available due to being proprietary but are available from the corresponding author on reasonable request. Other datasets analysed during the current study are available in the CEDA Archive, https://catalogue.ceda.ac.uk/uuid/049ff74d15b0ef759e966b275b8d39bb, KRILLBASE, http://dx.doi.org/10.5285/8b00a915-94e3-4a04-a903-dd4956346439, E.U. Copernicus Marine Service Information repositories, https://doi.org/10.48670/moi-00169 and https://doi.org/10.48670/moi-00047, and COMNAP, https://static1.squarespace.com/static/61073506e9b0073c7eaaf464/t/611497cc1ece1b43f0eeca8a/1628739608968/COMNAP_Antarctic_Station_Catalogue.pdf.

## Code availability

This study is entirely reproducible from code release v1.0 in repository https://github.com/aidanhunter-lab/CUPIDO-risk-map. The code is also available from 10.5281/zenodo.14170047.
