# Supplementary material for: Microplastic hotspots mapped across the Southern Ocean reveal areas of potential ecological impact
Source: Sci Rep. 2024 Dec 30;14:31599. doi: 10.1038/s41598-024-79816-y (PMC11685641; doi:10.1038/s41598-024-79816-y)
Supplement: Supplementary file 1 — Supplementary Information. [file 41598_2024_79816_MOESM1_ESM.pdf]

Microplastic hotspots mapped across the Southern Ocean reveal areas of potential ecological impact. Supplementary material

Table S1. Publications recording Southern Ocean microplastic samples.

Substrate	Region	Source
seawater	**AS**: AP (GSC)	Cózar et al. (2014)[1]
seawater	**AS**: AP, DP, LS (GSC)	Eriksen et al. (2014)[2]
seawater	**AS**: AP	Absher et al. (2019)[3]
seawater	**AS**: AP	Lacerda et al. (2019)[4]
seawater	**AS**: AP, SS	Jones-Williams et al. (2020)[5]
seawater	**AS**: AP	Alurralde et al. (2022)[6]
seawater	**PS**: RS, SoS	Cincinelli et al. (2017)[7]
seawater	**PS**: US, MS	Isobe et al. (2017)[8]
seawater	**PS & IS**: RS, SoS, US, MS, DS	Zhang et al. (2022)[9]
seawater	**CP**	Kuklinski et al. (2019)[10]
seawater	**CP**	Suaria et al. (2020)[11]
freshwater	**AS**: AP	González-Pleiter et al. (2020)[12]
sediment	**AS**: AP	Reed et al. (2018)[13]
sediment	**AS**: SS	Cunningham et al. (2020)[14]
sediment	**PS**: RS	Munari et al. (2017)[15]
ice	**AS**: AP	González-Pleiter (2021)[16]
ice	**PS**: MS	Kelly et al. (2020)[17]
seawater, freshwater	**AS & PS**: SS, DP, AP	Adventure Scientists (2022)[18]
seawater, freshwater, wastewater	**AS**: SS	Buckingham et al. (2022)[19]
seawater, sediment, ice, air	**AS**: AP, WS, SS, LS	Cunningham et al. (2022)[20]

AS = Atlantic Ocean sector: AP = Antarctic Peninsula, DP = Drake Passage, LS = Lazarev Sea, SS = Scotia Sea, WS = Weddell Sea
PS = Pacific Ocean sector: MS = Mawson Sea, RS = Ross Sea, SoS = Somov Sea, US = D'Urville Sea
IS = Indian Ocean sector: DS = Davis Sea
CP = circumpolar. GSC = from global sampling campaign

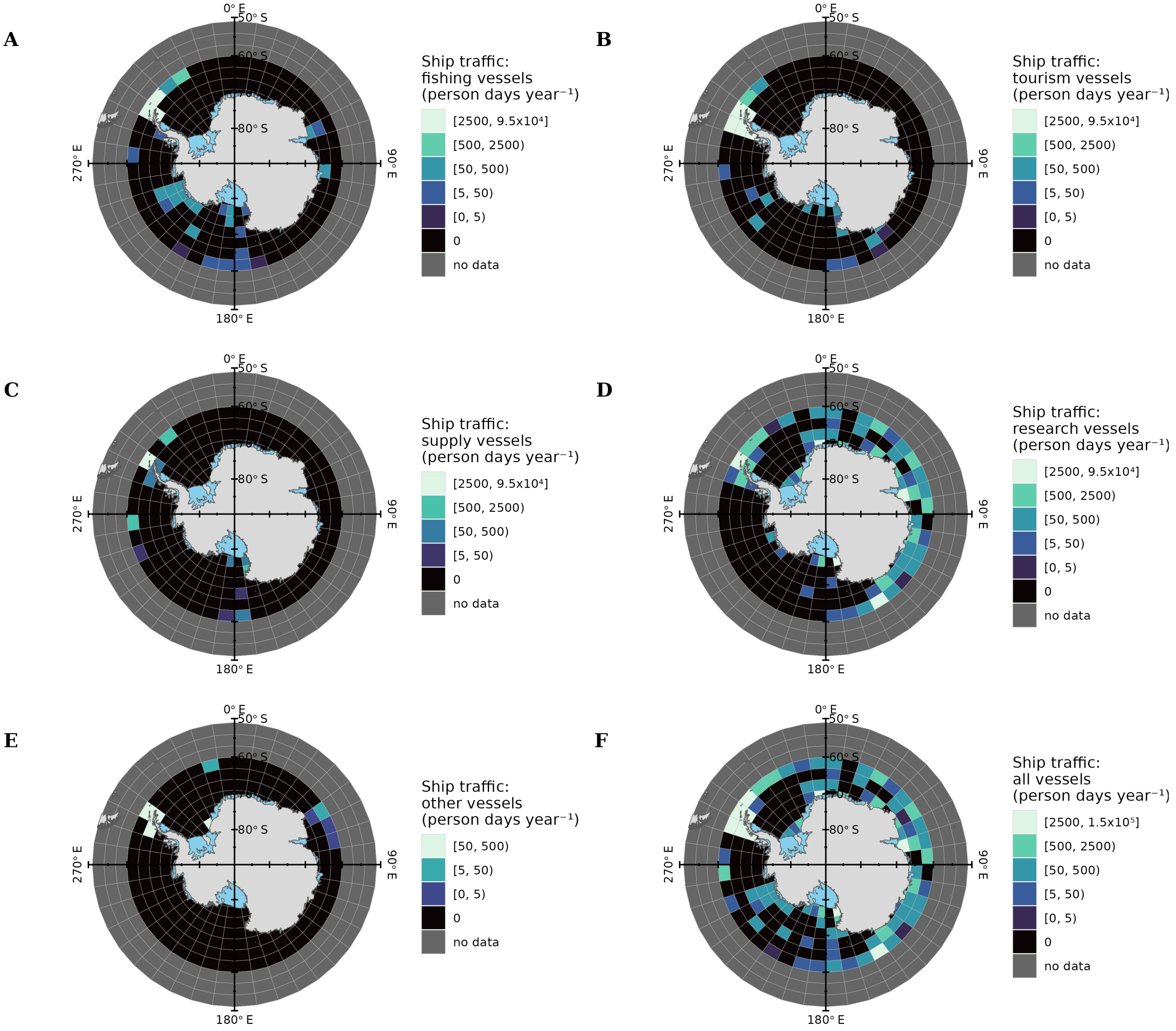

Figure S1. Maps of annual mean ship traffic derived from AIS data spanning 2014–2018. **A**–**E**: different vessel types. **F**: total ship traffic.

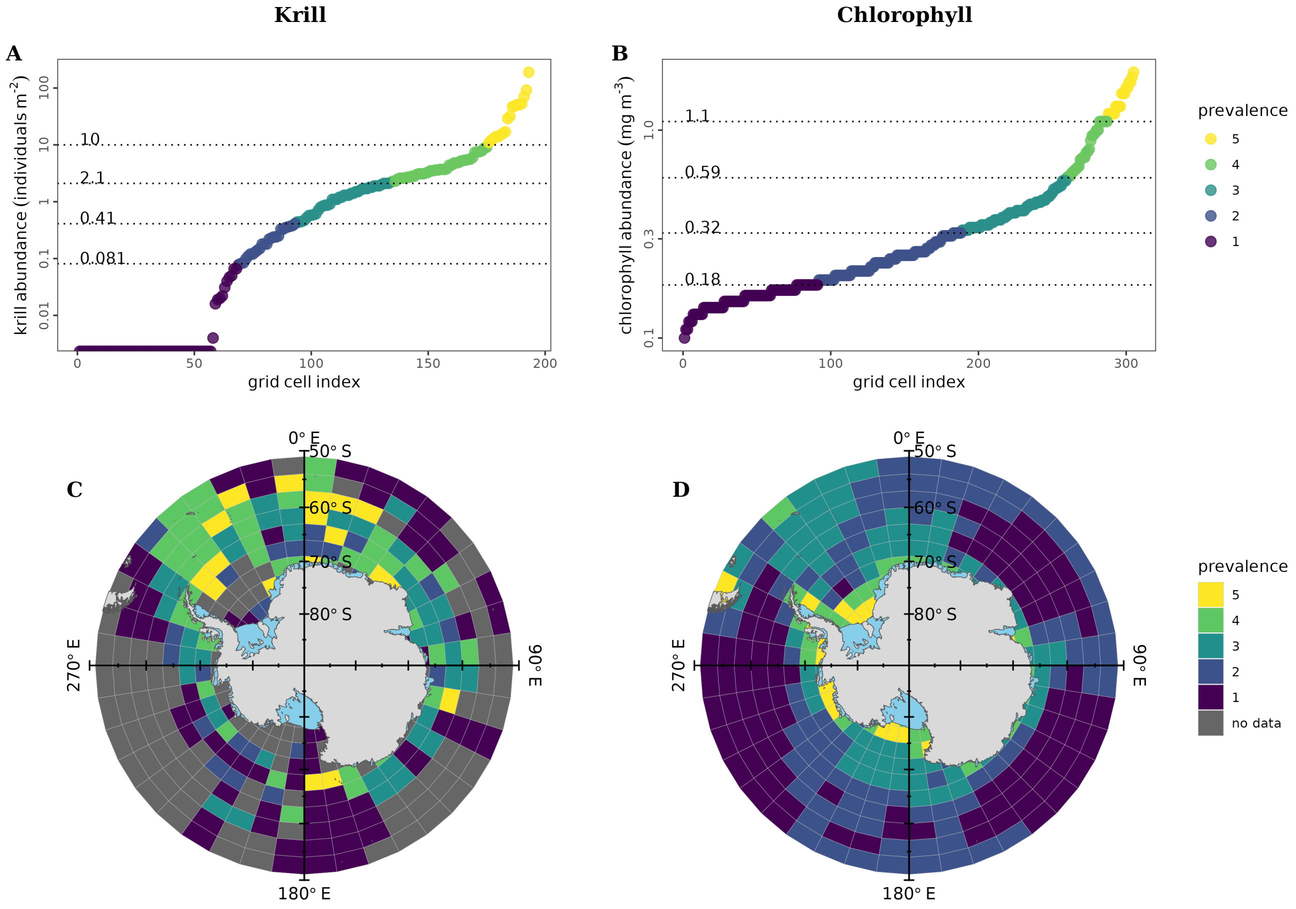

Figure S2. **A** and **B**: krill abundance and chlorophyll *a* concentration data grouped into five prevalence ranks. Numbers beside dotted lines are rank thresholds. **C** and **D**: maps of krill and chlorophyll *a* corresponding to these prevalence ranks.

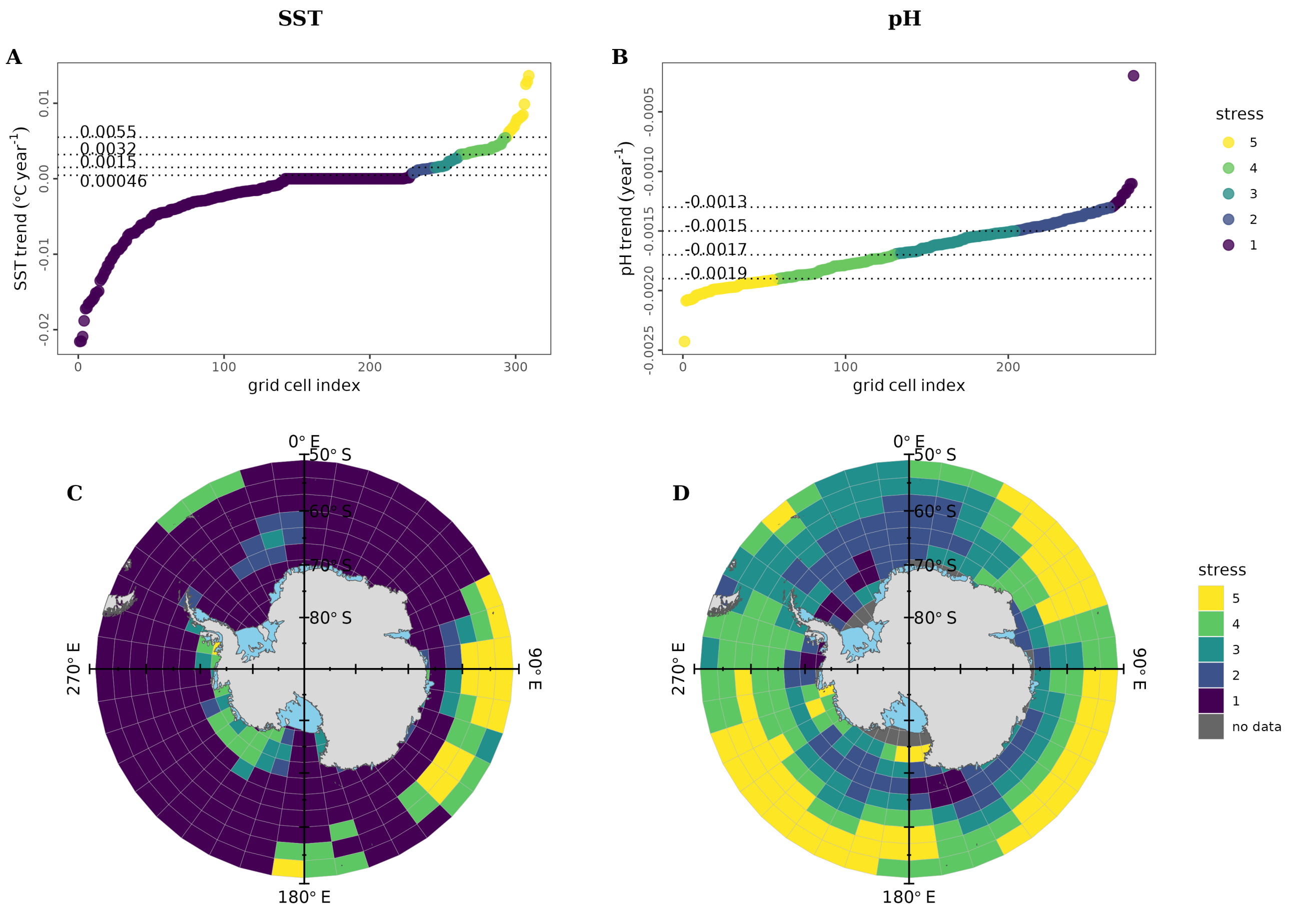

Figure S3. **A** and **B**: linear trends of sea surface temperature and pH grouped into five ranks indicating stress level. Numbers beside dotted lines are rank thresholds. **C** and **D**: maps of sea surface temperature and pH trends corresponding to these stress ranks.

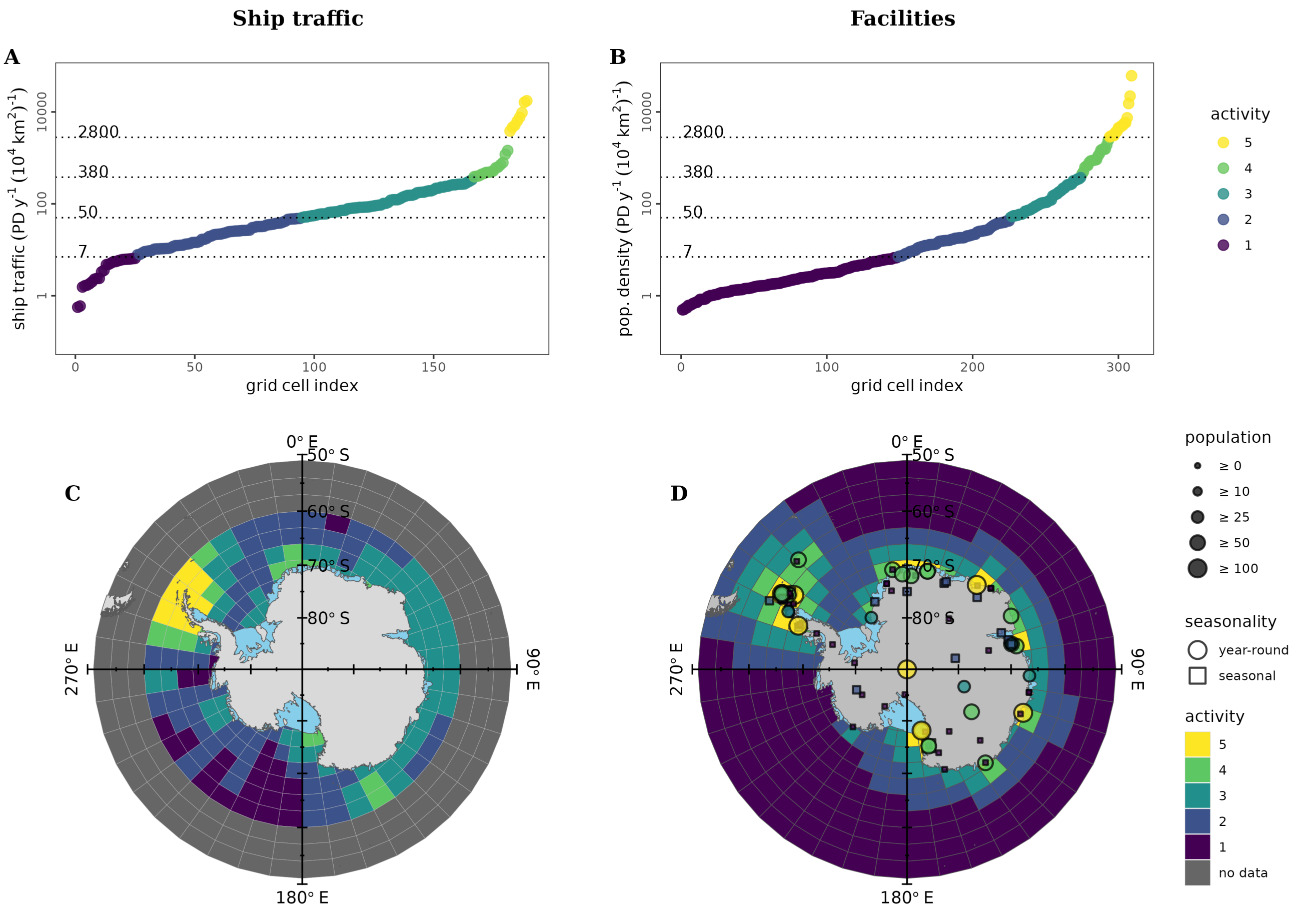

Figure S4. **A** and **B**: population density $\left(\text{person days year}^{-1}\ \left(10{,}000\ \text{km}^2\right)^{-1}\right)$ from ship traffic and facilities grouped into five ranks indicating human activity. Numbers beside dotted lines are rank thresholds. **C** and **D**: maps of ship traffic and facility population density corresponding to these activity rankings.

References

1. Cózar, A. *et al.* Plastic debris in the open ocean. *Proc. Natl. Acad. Sci.* **111**, 10239–10244, DOI: https://doi.org/10.1073/pnas.1314705111 (2014). https://www.pnas.org/content/111/28/10239.full.pdf.

2. Eriksen, M. *et al.* Plastic pollution in the world's oceans: More than 5 trillion plastic pieces weighing over 250,000 tons afloat at sea. *PLoS One* **9**, 1–15, DOI: https://doi.org/10.1371/journal.pone.0111913 (2014).

3. Absher, T. M. *et al.* Incidence and identification of microfibers in ocean waters in Admiralty Bay, Antarctica. *Environ. Sci. Pollut. Res.* **26**, 292–298, DOI: https://doi.org/10.1007/s11356-018-3509-6 (2019).

4. Lacerda, A. L. d. F. *et al.* Plastics in sea surface waters around the Antarctic Peninsula. *Sci. Rep.* **9**, 3977, DOI: https://doi.org/10.1038/s41598-019-40311-4 (2019).

5. Jones-Williams, K. *et al.* Close encounters — microplastic availability to pelagic amphipods in sub-Antarctic and Antarctic surface waters. *Environ. Int.* **140**, 105792, DOI: https://doi.org/10.1016/j.envint.2020.105792 (2020).

6. Alurralde, G. *et al.* Anthropogenic microfibres flux in an Antarctic coastal ecosystem: The tip of an iceberg? *Mar. Pollut. Bull.* **175**, 113388, DOI: https://doi.org/10.1016/j.marpolbul.2022.113388 (2022).

7. Cincinelli, A. *et al.* Microplastic in the surface waters of the Ross Sea (Antarctica): Occurrence, distribution and characterization by FTIR. *Chemosphere* **175**, 391–400, DOI: https://doi.org/10.1016/j.chemosphere.2017.02.024 (2017).

8. Isobe, A., Uchiyama-Matsumoto, K., Uchida, K. & Tokai, T. Microplastics in the Southern Ocean. *Mar. Pollut. Bull.* **114**, 623–626, DOI: https://doi.org/10.1016/j.marpolbul.2016.09.037 (2017).

9. Zhang, S. *et al.* Distribution characteristics of microplastics in surface and subsurface Antarctic seawater. *Sci. Total. Environ.* **838**, 156051, DOI: https://doi.org/10.1016/j.scitotenv.2022.156051 (2022).

10. Kuklinski, P. *et al.* Offshore surface waters of Antarctica are free of microplastics, as revealed by a circum-Antarctic study. *Mar. Pollut. Bull.* **149**, 110573, DOI: https://doi.org/10.1016/j.marpolbul.2019.110573 (2019).

11. Suaria, G. *et al.* Floating macro- and microplastics around the Southern Ocean: Results from the Antarctic Circumnavigation Expedition. *Environ. Int.* **136**, 105494, DOI: https://doi.org/10.1016/j.envint.2020.105494 (2020).

12. González-Pleiter, M. *et al.* First detection of microplastics in the freshwater of an Antarctic Specially Protected Area. *Mar. Pollut. Bull.* **161**, 111811, DOI: https://doi.org/10.1016/j.marpolbul.2020.111811 (2020).

13. Reed, S., Clark, M., Thompson, R. & Hughes, K. A. Microplastics in marine sediments near Rothera Research Station, Antarctica. *Mar. Pollut. Bull.* **133**, 460–463, DOI: https://doi.org/10.1016/j.marpolbul.2018.05.068 (2018).

14. Cunningham, E. M. *et al.* High abundances of microplastic pollution in deep-sea sediments: Evidence from Antarctica and the Southern Ocean. *Environ. Sci. Technol.* **54**, 13661–13671, DOI: https://doi.org/10.1021/acs.est.0c03441 (2020).

15. Munari, C. *et al.* Microplastics in the sediments of Terra Nova Bay (Ross Sea, Antarctica). *Mar. Pollut. Bull.* **122**, 161–165, DOI: https://doi.org/10.1016/j.marpolbul.2017.06.039 (2017).

16. González-Pleiter, M. *et al.* A pilot study about microplastics and mesoplastics in an Antarctic glacier. *The Cryosphere* **15**, 2531–2539, DOI: https://doi.org/10.5194/tc-15-2531-2021 (2021).

17. Kelly, A., Lannuzel, D., Rodemann, T., Meiners, K. M. & Auman, H. J. Microplastic contamination in east Antarctic sea ice. *Mar. Pollut. Bull.* **154**, 111130, DOI: https://doi.org/10.1016/j.marpolbul.2020.111130 (2020).

18. Adventure Scientists. Global microplastics initiative. URL https://www.adventurescientists.org/microplastics.html (2022).

19. Buckingham, J. W., Manno, C., Waluda, C. M. & Waller, C. L. A record of microplastic in the marine nearshore waters of South Georgia. *Environ. Pollut.* **306**, 119379, DOI: https://doi.org/10.1016/j.envpol.2022.119379 (2022).

20. Cunningham, E. M. *et al.* The transport and fate of microplastic fibres in the Antarctic: The role of multiple global processes. *Front. Mar. Sci.* **9**, DOI: https://doi.org/10.3389/fmars.2022.1056081 (2022).
